# CREB5 promotes the proliferation and self-renewal ability of glioma stem cells

**DOI:** 10.1038/s41420-024-01873-z

**Published:** 2024-02-28

**Authors:** Hyun-Jin Kim, Hye-Min Jeon, Don Carlo Batara, Seongsoo Lee, Suk Jun Lee, Jinlong Yin, Sang-Ik Park, Minha Park, Jong Bae Seo, Jinik Hwang, Young Joon Oh, Sung-Suk Suh, Sung-Hak Kim

**Affiliations:** 1 Department of Animal Science, College of Agriculture and Life Sciences, Chonnam National University, Gwangju, 61186 Republic of Korea; 2 Department of Cancer Biology, Lerner Research Institute, Cleveland Clinic, Cleveland, OH USA; 3 Gwangju Center, Korea Basic Science Institute (KBSI), Gwangju, 61186 Republic of Korea; 4 Department of Biomedical Laboratory Science, College of Health & Medical Sciences, Cheongju University, Chungbuk, 360764 Republic of Korea; 5 Henan-Macquarie Uni Joint Centre for Biomedical Innovation, Academy for Advanced Interdisciplinary Studies, Henan Key Laboratory of Brain Targeted Bio-nanomedicine, School of Life Sciences, Henan University, Kaifeng, 475004 Henan China; 6 Laboratory of Veterinary Pathology, BK21 FOUR Program, College of Veterinary Medicine, Chonnam National University, Gwangju, 61186 Republic of Korea; 7 Department of Biomedicine, BK21 FOUR Program, Health & Life Convergence Sciences, Biomedical and Healthcare Research Institute, Mokpo National University, Muan, 58554 Republic of Korea; 8 West Sea Fisheries Research Institute, National Institute of Fisheries Science, Incheon, 22383 Republic of Korea; 9 Technology Innovation Research Division, World Institute of Kimchi, Gwangju, 61755 Republic of Korea

**Keywords:** CNS cancer, CNS cancer, Cancer stem cells

## Abstract

Glioblastoma multiforme (GBM) is the most fatal form of brain cancer in humans, with a dismal prognosis and a median overall survival rate of less than 15 months upon diagnosis. Glioma stem cells (GSCs), have recently been identified as key contributors in both tumor initiation and therapeutic resistance in GBM. Both public dataset analysis and direct differentiation experiments on GSCs have demonstrated that CREB5 is more highly expressed in undifferentiated GSCs than in differentiated GSCs. Additionally, gene silencing by short hairpin RNA (shRNA) of CREB5 has prevented the proliferation and self-renewal ability of GSCs in vitro and decreased their tumor forming ability in vivo. Meanwhile, RNA-sequencing, luciferase reporter assay, and ChIP assay have all demonstrated the closely association between CREB5 and OLIG2. These findings suggest that targeting CREB5 could be an effective approach to overcoming GSCs.

## Introduction

Glioblastoma multiforme (GBM) is the most common and malignant brain tumor. Despite treatment options such as surgical resection, radiotherapy, and chemotherapy, the median survival rate following diagnosis is only 15 months [1, 2]. There is accumulating evidence that glioma stem cells (GSCs), also known as tumor initiating cells, play an important role in tumor recurrence and resistance to treatments [3, 4]. As such, studies that target the pro-tumorigenic features of GSCs are promising approaches that could lead to long term treatments for GBM.

CREB5, also known as CREB-BPA, belongs to the CREB (cAMP response element-binding protein) protein family, which is known to regulate cell growth, proliferation, and differentiation by binding to the cAMP-response elements through their domain bZIP DNA-binding and zinc-finger domains. Several studies have shown the diverse roles of CREB5, especially its involvement in tumor progression in various cancers [5–7]. For instance, it has been found that increased CREB5 expression is positively correlated with tumor cell invasion and a poor prognosis for cancer patients with epithelial ovarian cancer and hepatocellular carcinoma [8, 9]. Similarly, in colorectal cancer, in depth computational analysis has shown the involvement of CREB5 in the metastatic signal network, suggesting that it promotes metastasis and invasiveness by boosting MET expression and activating the HGF-MET signaling pathway [10]. On the other hand, in prostate cancer, CREB5 has been found to play a crucial role in promoting resistance to androgen receptor antagonists and androgen deprivation [11].

In this study, we demonstrate the role of CREB5 in glioblastoma, particularly in GSCs. We found that the knockdown of CREB5 in GSCs inhibited its proliferation and self-renewal activity in vitro and tumor forming ability in vivo. Interestingly, we found that OLIG2 is significantly downregulated by suppressing CREB5 expression. We also confirmed that CREB5 is highly expressed in GSCs and associated with poor survival in GBM patients. These findings suggest that overexpression of CREB5 is important in GSC progression.

## Results

### *CREB5* mRNA is expressed higher in the undifferentiated GSCs

**Fig. 1:**
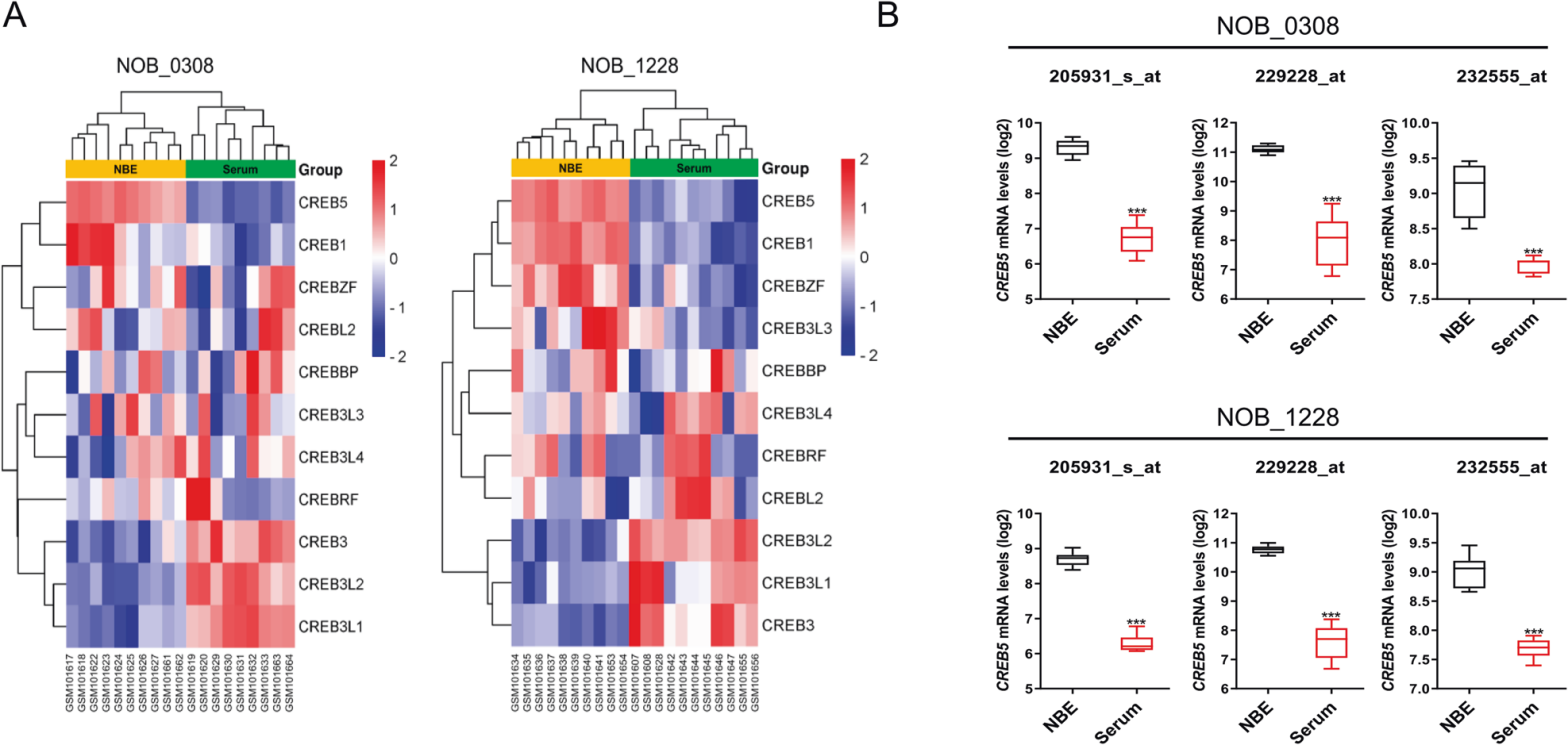
CREB5 mRNA is expressed higher in the undifferentiated GSC. **A**, **B** The comparison of CREB Family expression in GSCs cultured under NBE and Serum conditions according to multiple probes set of the GSE4536 dataset. **A** 0308 cells; **B** 1228 cells. Data are means ± SEM (NBE, *n* = 10 or 11; FBS, *n* = 11 or 10). ***p < 0.001.

Previous research has demonstrated that undifferentiated GSCs have stronger tumorigenic potential and self-renewal ability than differentiated GSCs [12]. We compared the expression pattern of genes belonging to the CREB family in undifferentiated and differentiated GSCs using a publicly available dataset (GSE4536). Among the CREB family, CREB5 expression was the highest in undifferentiated GSCs (“NBE” conditions: serum free DMEM/F12 media supplemented with basic FGF and EGF), while lowly expressed in differentiated GSCs (“Serum” conditions: DMEM/F12 media containing 10% FBS) (Fig. 1A, B). These findings suggest that CREB5 may play an essential role in the maintenance of GSCs.

### *CREB5* expression is correlated with poor prognosis

We also investigated the clinical relevance of CREB5 in GBM patients by examining the expression patterns and survival rates of the CREB family using the Rembrandt dataset. We first compared the expression of the CREB family genes in non-tumor and GBM patients. We found that several genes, including CREB5, were highly expressed in GBM (Supplementary Fig. S1A). Next, we analyzed the expression of CREB family genes according to glioma grade and observed that some genes, including CREB5, were highly expressed in grade IV GBM compared to grade II or III (Supplementary Fig. S1B). We also evaluated the survival rates of glioma patients based on the expression levels of CREB family genes. Our investigation revealed that only high expression of CREB5 was significantly associated with poor survival rates in GBM patients (Supplementary Fig. S1C). The link between high CREB5 expression and poor overall survival, as well as the increased expression of CREB5 in GBM, supports CREB5’s potential as a promising target in GBM.

### CREB5 is overexpressed in the classical subtype and highly expressed in the cellular tumor region and pseudopalisading cells around necrosis

GBM subtypes could be classified into proneural, classical, mesenchymal, and neural types based on genome wide analysis of mRNA expression in 300 GBM patient tissues [13]. We found that *CREB5* mRNA is highly expressed in the classical subtype in the Rembrandt dataset (Supplementary Fig. S2A). Moreover, in the classical GBM subtype, a correlation was confirmed between the CREB5 gene and genes important for stemness of glioma stem cells, such as OLIG2 and NES (Supplementary Fig. S2B). To identify the GBM anatomical region where CREB5 is preferentially expressed, we compared its expression patterns in the leading edge (LE), infiltrating tumor (IT), cellular tumor (CT), perinecrotic zone (PNZ), pseudopalisading cells around necrosis (PAN), hyperplastic blood vessels in cellular tumors (HBV), and microvascular proliferation (MVP) using the Ivy Glioblastoma Atlas Project dataset. We found that *CREB5* is highly enriched in the CT and PAN regions (Supplementary Fig. S2C).

### *CREB5* is highly expressed in GSCs

**Fig. 2:**
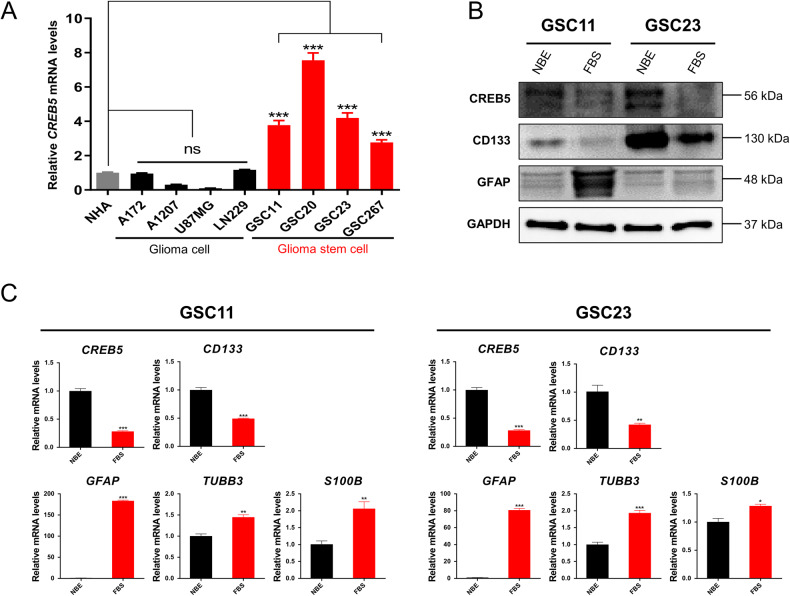
CREB5 is expressed highly expressed in GSC. **A** RT-qPCR analysis of CREB5 mRNA expression in NHA, non-stem cell glioma cells, and GSCs. **B** Western blot analysis of CREB5 protein in GSCs and differentiated GSCs. **C** RT-qPCR analysis of mRNA expressions in GSCs and differentiated GSCs. Data are means ± SEM (*n* = 3). *p < 0.05, **p < 0.01, ***p < 0.001.

To validate the expression patterns of CREB5 in gliomas, we analyze the mRNA expression of CREB5 in glioma cell lines. We found that GSCs had considerably higher levels of CREB5 expression when compared to non-stem cell glioma cells and normal human astrocytes (NHA) (Fig. 2A). Next, we cultured GSCs in FBS containing media for seven days to generate differentiated GSCs. The expression of stem cell markers decreased substantially in differentiated GSCs, while differentiation markers were significantly elevated. Also, differentiated GSCs had lower CREB5 expression than their undifferentiated counterparts (Fig. 2B, C). Based on these results, we suggest that the expression of CREB5 is significantly elevated in GSCs.

### Suppressing of CREB5 inhibits proliferation and self-renewal ability in GSCs

**Fig. 3:**
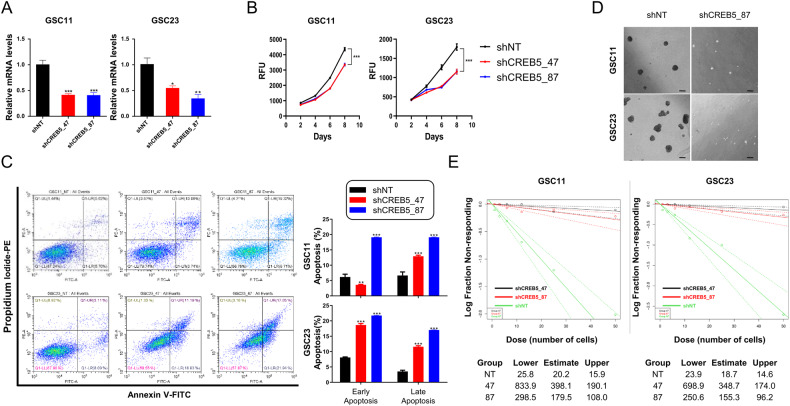
Suppressing of CREB5 inhibits proliferation and self-renewal ability in GSCs. **A** RT-qPCR analysis showing CREB5 knockdown after transduction with shRNA in GSCs. **B** Cell proliferation shCREB5 treated GSCs. **C** Annexin-V/propidium iodide staining of GSCs after transduction with shCREB5. **D** Representative images showing cell spheres. Scale bars = 100 μm. **E** An in vitro limiting dilution assay using gradually decreasing cell seeding density shows the cell sphere forming ability of GSCs transduced with shCREB5. Data are means ± SEM (*n* = 3). *p < 0.05, **p < 0.01, ***p < 0.001.

We next investigated the role of CREB5 in the proliferation and self-renewal ability of GSCs. Upon silencing the expression of CREB5, we observed a decrease in the proliferation of GSC11 and GSC23 cells (Fig. 3A, B). The Annexin V/PI assay also demonstrated that inhibition of CREB5 expression caused a shift in the distribution of GSCs towards apoptotic or necrotic regions (Fig. 3C). On the other hand, we observed that a decrease in CREB5 expression led to the suppression of GSC sphere growth (Fig. 3D). To ascertain the effects of decreased CREB5 expression on the self-renewal activity of GSCs, we carried out the serial limiting dilution assay. We confirmed a significant reduction in the characteristic of stem cell in GSCs when CREB5 expression was inhibited (Fig. 3E). These findings indicate that inhibition of CREB5 expression leads to decreased proliferation and self-renewal ability of GSCs.

### The inhibition of CREB5 reduces the tumorigenic potential of GSCs in vivo

**Fig. 4:**
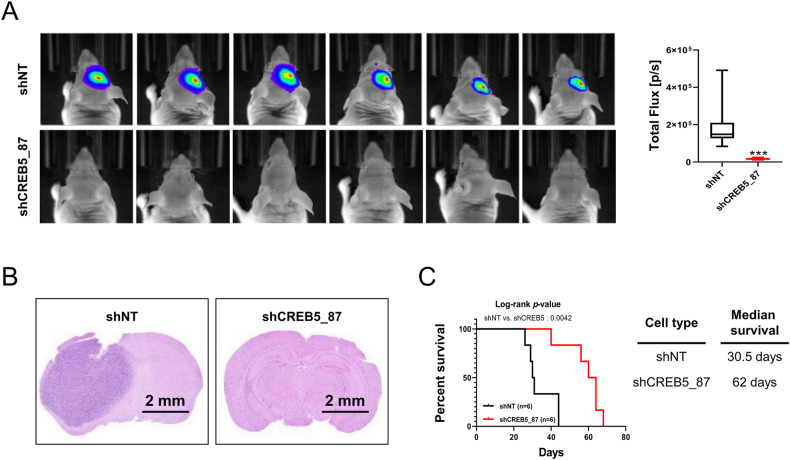
The inhibition of CREB5 reduces the tumorigenic potential of GSCs in vivo. **A** In vivo bioluminescent imaging was performed on nude mice bearing intracranial xenografts derived from shNT (Non-target) and shCREB5 transduced GSC11. Data are means ± SEM (*n* = 6). ***p < 0.001. **B** Representative images of H&E stained coronal sections of tumor bearing brains harvested after implantation of shNT and shCREB5 treated GSC11. Scale bars represent 2 mm. **C** Kaplan–Meier survival curves of nude mice bearing intracranial tumors derived from shNT and shCREB5 treated GSC11.

Using an in vivo orthotopic xenograft model, we evaluated the tumorigenic potential of CREB5. After suppressing the expression of CREB5 using shCREB5 in GSC11 cells, we noticed a significant decrease in the tumorigenic potential of knockdown cells compared to shNT(Non-target) control after injection into the brains of nude mice (Fig. 4A, B). We also observed there is a significant increase in mouse survival when inoculated with CREB5 knockdown GSCs (Fig. 4C). These data suggest that CREB5 could play an important role in the tumorigenic potential of GSCs in vivo.

### RNA-Sequencing reveals pathways and genes downregulated by shCREB5

**Fig. 5:**
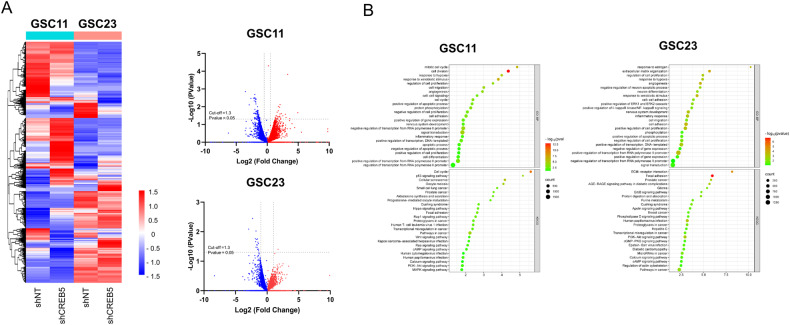
RNA-Sequencing reveals pathways and genes downregulated by shCREB5. **A** Heat map and volcano plots of transcriptional regulation patterns of shNT and shCREB5 transduced GSCs. **B** Dot plots present enrichment pathways of genes downregulated with CREB5 in shCREB5 transduced GSCs. Data are means ± SEM.

To understand the mechanism of how CREB5 regulates GSCs at the transcriptional level, we performed an RNA-sequencing analysis (Fig. 5A). KEGG pathway and Gene Ontology Biological Process analysis on the RNA-sequencing data revealed that upon CREB5 knockdown, the expression of genes that are related to multiple cancer stem cell associated signaling pathways, and cell cycle pathway were also decreased (Fig. 5B). These findings suggest the importance of CREB5 expression in the regulation of pathways that are important in GSC progression.

### CREB5 binds to the AP-1 sites within the OLIG2 promoter

**Fig. 6:**
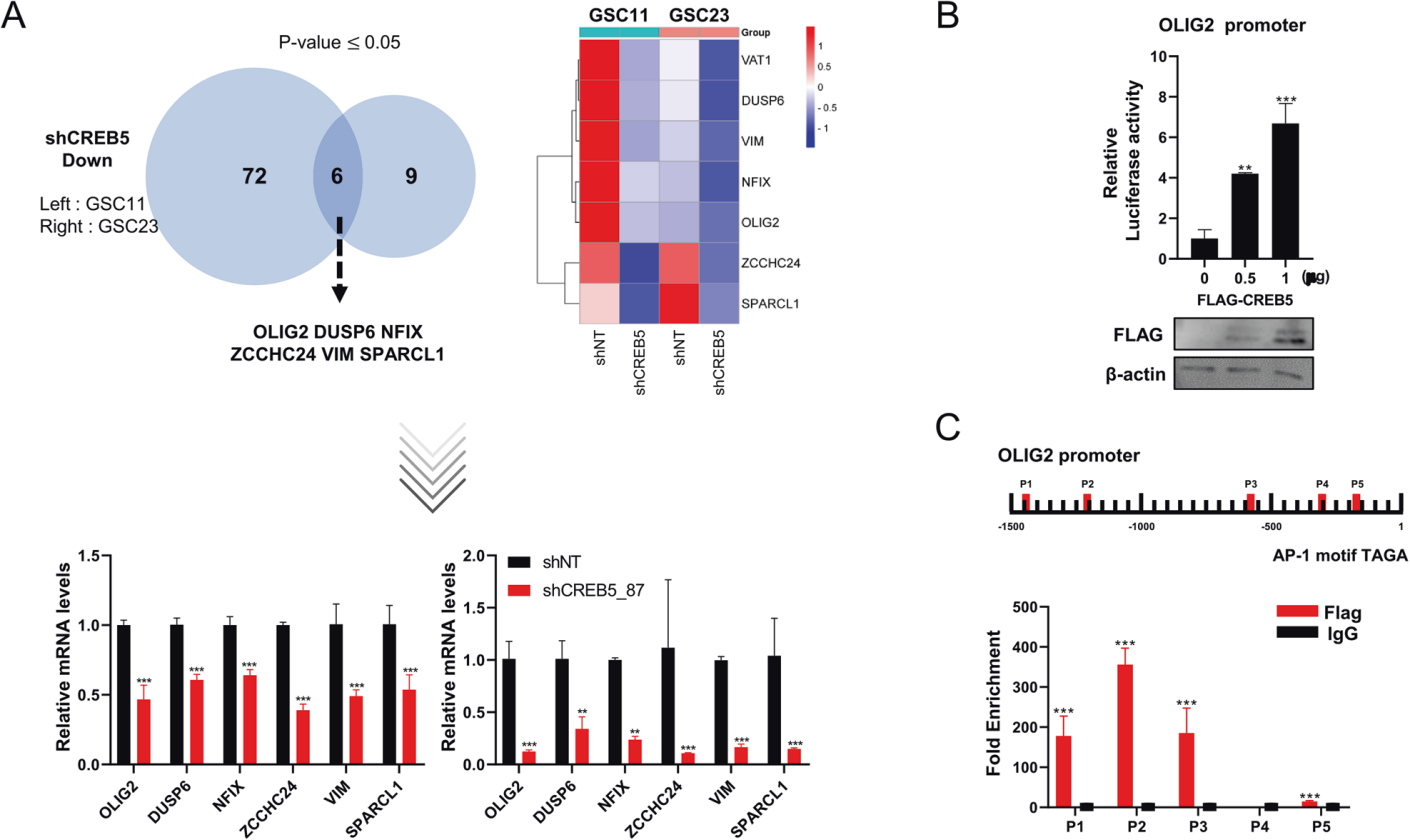
CREB5 binds to the AP-1 sites within the OLIG2 promoter. **A** RT-qPCR validation of downregulated genes after CREB5 inhibition. **B** Luciferase assay using OLIG2 promoter after indicated treatment. **C** ChIP analysis of CREB5 binding to the OLIG2 promoter in HEK293T. Data are means ± SEM (*n* = 3). *p < 0.05, **p < 0.01, ***p < 0.001.

Next, we identified downstream genes that are concomitantly decreased upon CREB5 inhibition in GSCs. The genes such as OLIG2, DUSP5, NFIX, ZCCHC24, VIM, and SPARCL1 were concurrently downregulated in both GSC11 and GSC23 cells (Fig. 6A). OLIG2 is a gene involved in central nervous system development, and there are reports that this gene plays an important role in the maintenance of brain tumors, especially GSCs [14–16]. Accordingly, OLIG2 is highly expressed in GSCs and that cell proliferation is suppressed when it is inhibited [17]. Therefore, taking into account the fact that CREB5 is a transcription factor and the downregulation of OLIG2 at the transcriptional level, we investigated whether CREB5 influences the activity of the OLIG2 promoter using a luciferase reporter assay (Fig. 6B). As a result, we observed an increase in OLIG2 promoter activity upon overexpression of CREB5 in HEK293T cells in a concentration dependent manner (Fig. 6B). Furthermore, we performed chromatin immunoprecipitation (ChIP) assay and identified multiple AP-1 sites within the OLIG2 promoter where CREB5 protein could potentially bind (Fig. 6C) [10]. Consistently, we found a positive correlation between CREB5 and OLIG2 expression in GBM by utilizing various patient datasets (Supplementary Fig. S3). These findings confirm that OLIG2 is a transcriptional target of CREB5.

## Discussion

Among CREB family proteins, CREB5 is specifically highly expressed and strongly associated with poor patient survival in GBM. Due to its differential abundance in the GSC population, we investigated the function of CREB5 by suppressing its expression and examining the mechanism to regulate GSC. Interestingly, CREB5 knockdown significantly inhibits GSCs’ survival, self-renewal activity in vitro, and tumorigenic potential in a mouse xenograft model. RNA sequencing analysis revealed CREB5 modulates a variety of signaling pathways including cell cycle, PI3K-Akt, and focal adhesion. Among the putative target genes of CREB5, we have focused on OLIG2 due to its clinical relevance in GBM.

We then hypothesize the role CREB5-OLIG2 axis in GSCs as follows: 1) as a central nervous system (CNS) restricted transcription factor, it plays an essential role in glial progenitor proliferation [18–20]; (2) it is widely expressed in gliomas and plays a critical role in gliomagenesis and tumor phenotype plasticity; and [15–17, 21–23] (3) recently, OLIG2 has been identified as a core transcription factor, along with SOX2, SALL2, and POU3F2, that reprograms differentiated GBM cells into GSCs [14]. Therefore, we postulate that CREB5 might regulate OLIG2 to be involved in the maintenance of GSCs.

Meanwhile, we found that CREB5 expression is strongly associated with poor patient survival and upregulated in GBM classical subtype in publicly available datasets. The classical GBM subtype is characterized by a high frequency of EGFR gene amplification and mutation [13]. Aberrant EGFR amplification or mutation may influence the activation of signaling networks that promotes irregular cell growth and survival, ultimately driving tumor progression [24–26]. EGFR signaling pathways interact with downstream effectors, such as the RAS/RAF/MEK/ERK and PI3K/Akt/mTOR pathways [27, 28] vital for cell communication and interaction, regulating basic cell processes like growth, survival, and differentiation. Interestingly, after inhibiting CREB5 expression, we found that the genes associated with PI3K-Akt signaling were decreased in both GSC11 and GSC23 cells. This suggests that there might be a negative feedback loop that starts from CREB5, suppressing the PI3K-Akt signaling pathway.

On the other hand, RNA-seq data showed that four other genes (DUSP6, NFIX, VIM, and SPARCL1) associated with cancer were also downregulated in CREB5 knockdown cells. The DUSP6 (dual specificity phosphatase 6) is highly expressed in GBM and its expression is associated with poor patient survival [29]. Findings also show that exogenous overexpression of DUSP6 increases tumor growth as well as resistance to cisplatin mediated cell death in both in vitro and in vivo experiments [30]. Interestingly, DUSP6 was identified as hub genes and anti-cancer compounds can be developed by inhibiting the interaction of ERK2 and DUSP6 [29]. Similarly, the NFIX (nuclear factor IX) was also found to be upregulated in GBM and transcriptionally upregulates Ezrin, a protein that crosslinks the cytoskeleton and plasma membrane. Suppression of NFIX in GBM cells impairs cell proliferation and migration in vitro and increases the survival rate in mouse orthotopic xenograft models [31]. It was found that NFIX binds to the promoter of the Go-Ichi-Ni-San 1 (GINS1) gene, regulating GBM cell proliferation. NFIX inhibition in a GINS1 dependent manner, increases the sensitivity of GBM cells to DNA damage inducing agents, such as doxorubicin and temozolomide [32]. VIM (Vimentin) is highly expressed in gliomas compared to non-tumor tissues. Reports show that VIM inhibition reduces the migration ability in GBM cells (U87MG, U251, and U373) [33]. The anti-vimentin nanobody (Nb79) considerably diminishes the invasion capacity of both the differentiated GBM cell line (U87MG) and the GSC line (NCH421k). Recently, the invasion inhibitory effect of Nb79 was also observed on U87MG and NCH421k in vitro and in vivo in zebrafish embryos [34]. Lastly, proteomics analysis revealed that SPRL1 (Sparc like protein 1), the protein encoded by secreted, acidic, and rich in cysteine like 1 (SPARCL1), is highly expressed in mouse brain tumor xenograft models. Also, the elevated SPRL1 expression is associated with high grade glioma samples [35]. Findings show that overexpression of SPARCL1 promotes neo-angiogenesis in intracranial xenografts derived from proneural and mesenchymal GSCs and endow the angio-architectural pattern in patients. Furthermore, SPARCL1 triggers a notable rise in activated microglia, which corresponds with the augmentation of angiogenesis [36]. As such, additional studies on these other genes and their association with CREB5 expression are important and could provide insights, especially on their role in GBM progression. Meanwhile, in recent studies it was reported that high glucose can activate the PI3K/Akt/CREB5 signaling pathway, resulting in excessive proliferation and migration of vascular smooth muscle cells [37]. Identifying the upstream regulatory elements that affect CREB5 expression and activity in GBM is also necessary.

In conclusion, our data suggest that CREB5 plays a critical role in maintaining GSCs by regulating OLIG2. Targeting CREB5 may be a promising approach not just eradicating GSCs but also improving GBM treatment.

## Materials and methods

### Cell culture

Normal human astrocytes (NHA) were cultured in an astrocyte medium (ScienCell Research Laboratories, USA) supplemented with 10% fetal bovine serum (FBS; Gibco, USA), 1% astrocyte growth supplement (AGS; ScienCell Research Laboratories, USA), and 1% penicillin/streptomycin (P/S; Welgene, Republic of Korea). The glioma cell lines (A172, A1207, U87MG, LN229) and HEK293T were cultured in Dulbecco’s modified medium (DMEM/F12; Welgene, Republic of Korea) supplemented with 10% FBS, and 1% P/S. The glioma stem cells (GSC11, GSC20, GSC23, GSC267) obtained from the University of Texas MD Anderson Cancer Center [38] were cultured in a Neurobasal Medium (NBE) comprising of DMEM/F12, 1% P/S, 2% B27 (Gibco, Thermo Fisher Scientific, USA), epidermal growth factor (EGF; 20 ng/ml; R&D Systems, USA), and basic fibroblast growth factor (bFGF; 20 ng/ml; R&D Systems, USA). All GSC cell lines have been authenticated and are Mycoplasma-free. (COSMO GENETECH, Republic of Korea). All cells were maintained at 37 °C with 5% CO_2_.

### Quantitative reverse transcription-PCR (RT- qPCR)

Total RNA was extracted using RiboEX reagent (GeneAll, Republic of Korea) and purified with the HybridR kit (GeneAll, Republic of Korea) according to the manufacturer’s instructions. The RevertAid First Strand cDNA Synthesis Kit (Thermo Fisher Scientific, MA, USA) was used to synthesize cDNA from 500 ng of total RNA. Quantitative Real Time PCR (qPCR) was performed using the TB Green Premix Ex Taq (Tli RNaseH Plus; Takara Korea Biomedical Inc., Korea) on a BioRad Laboratories CFX96 realtime polymerase chain reaction detection system (CA, USA). The cycle threshold (Ct) values from qPCR results were analyzed using the 2^−ΔΔCt^ method. The following primer sequences (5′ to 3′) were used for qPCR: 18S (loading control): forward F, CAGCCACCCGAGATTGAGCA and reverse R, TAGTAGCGACGGGCGGTGTG; CREB5: F, GAGCGACAAATGTCAGTGAACTCC and R, TGAGTCAATGCAGCCTTCAACC; CD133: F, CAGGTAAGAACCCGGATCAA and R, TCAGATCTGTGAACGCCTTG; GFAP: F, GGAACATCGTGGTGAAGACC and R, AGAGGCGGAGCAACTATCCT; TUBB3: F, AGTGTGAAAACTGCGACTGC and R, ACGACGCTGAAGGTGTTCAT; S100B: F, TCAAAGAGCAGGAGGTTGTG and R, TCGTGGCAGGCAGTAGTAAC; OLIG2: F, ATGCACGACCTCAACATCGCCA and R, ACCAGTCGCTTCATCTCCTCCA; DUSP6: F, CTCGGATCACTGGAGCCAAAAC and R, GTCACAGTGACTGAGCGGCTAA; NFIX: F, CGATGACAGTGAGATGGAGAGC and R, GCAGAAGTCCAGCTTTCCTGAC; ZCCHC24: F, CAGGAGTGCATCAAGTGCCACA and R, AGGACCTTGCACTTCTCGCAGA; VIM: F, AGGCAAAGCAGGAGTCCACTGA and R, ATCTGGCGTTCCAGGGACTCAT; SPARCL1: F, GTGAAGGCAACATGAGGGTGCA and R, GTTGGAGGACAAGTCACTGGATC.

### Western blot analysis

The cells were lysed using a combination of RIPA buffer (Thermo Scientific, CA, USA) and phosphatase inhibitor cocktail 2 obtained from APExBIO Technology LLC (Houston, Texas, USA). To determine the protein content of the lysate, we used the BCA Protein Assay Kit from Thermo Scientific (CA, USA). The proteins were fractionated using SDS polyacrylamide gel electrophoresis with a separation matrix comprising 10 and 15% polyacrylamide. The proteins were then transferred onto a polyvinylidene fluoride (PVDF) membrane. To prevent the non-specific binding of the primary antibodies, we blocked the PVDF membranes using a 5% skim milk solution in PBST for 1 h at room temperature. Following this, the membranes were incubated with the relevant primary antibodies overnight at 4 °C with gentle shaking. After primary antibody incubation, the membrane was washed with PBST. Subsequently, the membrane was developed by incubating with a secondary antibody for 1 h at room temperature. Following the secondary antibody incubation, the PVDF membranes were washed with PBST. The visualization of the membranes was carried out using chemiluminescence following the manufacturer’s instructions provided by Invitrogen (CA, USA). Antibodies used were: CREB5 (ab168928; Abcam, Cambridge, UK), CD133 (ab278053; Abcam, Cambridge, UK), GFAP (840001; BioLegend, CA, USA), GAPDH (14C10; Cell Signaling, MA, USA), FLAG (F1804, Sigma Aldrich, MO, USA), and β-actin (A5316; Sigma Aldrich, MO, USA).

### Dataset analysis using public datasets

The mRNA expression levels of the CREB family were compared among various GSC cultures using the Gene Expression Omnibus (GSE4536) datasets. The gene expressions were normalized using the dChip invariant method, and the PM-MM difference model was used to calculate the expression values. Furthermore, the analysis of CREB5 gene expression profiles and correlation analysis with patient survival and other GSC related genes was conducted using the Rembrandt dataset obtained from the Gliovis website (http://gliovis.bioinfo.cnio.es/).

### Lentiviral infection for CREB5 knockdown

Lentiviral vectors were used to express non-targeting shRNA (shNT) and shRNA constructs targeting CREB5 (TRCN0000271247, TRCN0000013487; Sigma Aldrich, MO, USA) to suppress CREB5 expression. To package the lentivirus, 293FT cells (Invitrogen, CA, USA) were transfected using the CalPhos Mammalian Transfection Kit (Takara Bio, Tokyo, Japan). We used pMD2.G and psPAX2 as packaging plasmids. The lentivirus was harvested 72 h after transfection and concentrated 100 fold with the Lenti-X concentrator (Takara Bio, Tokyo, Japan). Lentivirus infection was carried out following the manufacturer’s protocol.

### Cell viability

The cell viability of GSCs after shRNA transduction was assessed using the alamarBlue® cell viability assay (Invitrogen, CA, USA). The shRNA transduced GSCs were seeded at a density of 3000 cells/well (*n* = 6) in 96 well plates and incubated for 72 h. Then, 10 μl of alamarBlue reagent was added to each well and incubated for an additional 6 h. After the incubation period, fluorescence was measured at a wavelength of 590 nm using a Synergy HTX Multi Mode Reader (VT, BioTek Instruments Inc., USA).

### In vitro limiting dilution assay

An in vitro limiting dilution assay was performed to assess the tumor sphere formation ability of shRNA transduced GSCs. The shRNA transduced GSCs were seeded in decreasing cell numbers (50, 25, 12, 6, 3, and 1 cell/well; *n* = 30) in a 96 well plate. Cells were supplemented with 10 µl growth media every after 3 days and maintained until 14 days. The plates were examined under a light microscope at the end of the incubation period for tumor sphere formation. Cell clusters measuring more than 20 μm in diameter were considered to be positive wells. The frequency of tumor sphere formation ability of the GSCs was determined using the Extreme Limiting Dilution Analysis (ELDA) software, which can be accessed at http://bioinf.wehi.edu.au/software/elda [39]

### Annexin-V and propidium iodide staining

GSC11 and GSC23 were treated with shNT or shCREB5 for 72 h. After that, the cells were collected and washed with cold PBS. The cells were then incubated with Annexin-V and propidium iodide (Invitrogen, CA, USA) at room temperature for 15 min and analyzed using flow cytometry (Beckman Coulter, CA, USA).

### RNA-sequencing

GSCs were transduced with either shNT or shCREB5 and cultured in a 6 well plate for 2 days before harvesting at a density of 5 ×10^5^ cells. After washing with PBS, the cell pellets were resuspended in TRIzol Reagent (Invitrogen, CA, USA) and stored at −80 °C. RNA extraction, library preparation, and sequencing were outsourced to LAS Co., Ltd. (Gimpo, Republic of Korea).

### In vivo orthotopic implantation

For in vivo orthotopic implantation, shNT or shCREB5 treated GSCs were intracranially injected into the brains of BALB/c nude mice (female, 5 weeks old) at a concentration of 5 ×10^5^ cells using a stereotaxic instrument at coordinates of 2 mm right and 1.0 mm anterior of the bregma (randomization of *n* = 6 mice per group). Mice that lost more than 30% of their body weight were sacrificed by established ethical protocols. The overall survival curves were generated using the Kaplan–Meier method. To perform tissue histological analysis, one mouse from both the control and experimental groups was sacrificed simultaneously one month after the GSC injection. All experiments involving mice were carried out in compliance with the applicable standards and regulations of the Republic of Korea government and the institution and were authorized by the Animal Care Committee of Chonnam National University (CNUIACUC-YB-2021-99).

### Kyoto Encyclopedia of Genes and Genomes (KEGG) and Gene Ontology Biological Process (GO-BP) pathway analysis

To identify the genes that were downregulated by shCREB5, we performed KEGG pathway and GO-BP analysis using the DAVID website [40, 41].

### Luciferase reporter assay

To amplify the OLIG2 promoter fragment, Human Genomic DNA (Promega, WI, USA) was used as the source material. The OLIG2 promoter region was obtained by PCR amplification and then ligated into the pGL3-Basic vector (Promega, WI, USA) following the manufacturer’s instructions. HEK293T cells were transfected in a 24 well plate at 70% confluence using Lipofectamine 2000 (Thermo Scientific, CA, USA). After 48 h, luciferase activity was measured using the dual luciferase reporter system (Promega, WI, USA) and normalized to the expression of *Renilla* luciferase. All experimental procedures were performed in triplicate.

### Chromatin immunoprecipitation (ChIP) assay

To crosslink chromatin associated proteins to DNA, approximately 1 × 10^7^ cells were treated with 37% formaldehyde for 10 min in a 100 mm culture dish. Then, glycine was added to quench the unreacted formaldehyde. The cells were collected in 1 ml of sodium dodecyl sulfate lysis buffer with 1 μl of protease inhibitor cocktail added. The lysates were sonicated for 15 mins using 30 secs pulses at 30% output to fragment the DNA into pieces ranging from 200 to 1000 base pairs. After clearing the lysates by centrifugation at 13,000 rpm for 10 mins at 4 °C, 200 μl of the lysates were mixed with 1800 μl of dilution buffer, and 60 μl of protein A/G agarose (Thermo Scientific, CA, USA) was added. The mixture was then incubated at 4 °C for 1 h to preclear the chromatin. The precleared lysates were then incubated overnight at 4 °C with rotation with Flag antibody or normal mouse immunoglobulin G as a negative control. Immunoprecipitation of the DNA-protein complexes was performed using 60 μl of protein A/G agarose (Thermo Scientific, CA, USA) for 1 h at 4 °C, followed by isolation of the DNA. The human OLIG2 promoter was amplified by q-PCR, and all ChIP assays were performed three times. The following primer sequences : P1: F, GCATCCGAGATCTGCAGAAACAA and R, TACAGGCAGCCACCTGTCTC; P2: F, TGGGTGAATGCATCCGTACCT and R, TTACCGATTGCAGGCTGGCT; P3: F, GCCAAATGCCCACGTGTTGA and R, CAGGATCCGGGGCTGGG; P4: F, TGACCACGTTCCCTTTCTCCCT and R, CCTCCCTGCGCACAACCAATG; P5: F, CCCAAGAATCTCCCGGCCAC and R, AAGCTGATGTCATCCGGGCT.

### Statistical analysis

For statistical analysis, we used Microsoft Excel and GraphPad Prism Ver.9.0 software. To assess the significance between the two groups, we performed Student’s *t* test in GraphPad Prism. To evaluate the statistical significance among multiple groups, we conducted a one-way analysis of variance (ANOVA), followed by Tukey’s multiple comparison test. We considered the *p*-value less than 0.05 as statistically significant.

### Supplementary information

Supplementary Figures legend

Supplementary Figure 1

Supplementary Figure 2

Supplementary Figure 3

Original Data File

## Data Availability

The datasets generated during and/or analyzed during the current study are available in the NCBI SRA PRJNA954560 repository.
