# Supplementary material for: CREB5 promotes the proliferation and self-renewal ability of glioma stem cells
Source: Cell Death Discov. 2024 Feb 28;10:103. doi: 10.1038/s41420-024-01873-z (PMC10901809; doi:10.1038/s41420-024-01873-z)
Supplement: Supplementary file 1 — Supplementary Figures legend [file 41420_2024_1873_MOESM1_ESM.docx]

**Fig. S1 CREB5 expression is correlated with poor prognosis. A** Comparison of CREB family mRNA expression in non-tumor and GBM. **B** Comparison of mRNA expression of CREB family by glioma grade. **C** Overall survival rate of glioma patients and CREB family expression. All data sources are from the Rembrandt dataset. Data are means ± SEM **p* < 0.05, ***p* < 0.01, ****p* < 0.001.

**Fig. S2 CREB5 is overexpressed in the classical subtype and highly expressed in the cellular tumor region and pseudopalisading cells around necrosis. A** The correlation analysis between CREB5 expression was performed with classical, mesenchymal, proneural, and neural GSC markers in Rembrandt dataset. **B** Comparison of CREB5 mRNA expression in different regions of GBM. Data are means ± SEM ****p* < 0.001.

**Fig. S3 CREB5 is overexpressed in the classical subtype and highly expressed in the cellular tumor region and pseudopalisading cells around necrosis.** Correlation analysis of CREB5 and OLIG2 mRNA expression in multiple GBM datasets. Data are means ± SEM.
