# Supplementary figures and images for: CREB5 promotes the proliferation and self-renewal ability of glioma stem cells
Source: Cell Death Discov. 2024 Feb 28;10:103. doi: 10.1038/s41420-024-01873-z (PMC10901809; doi:10.1038/s41420-024-01873-z)

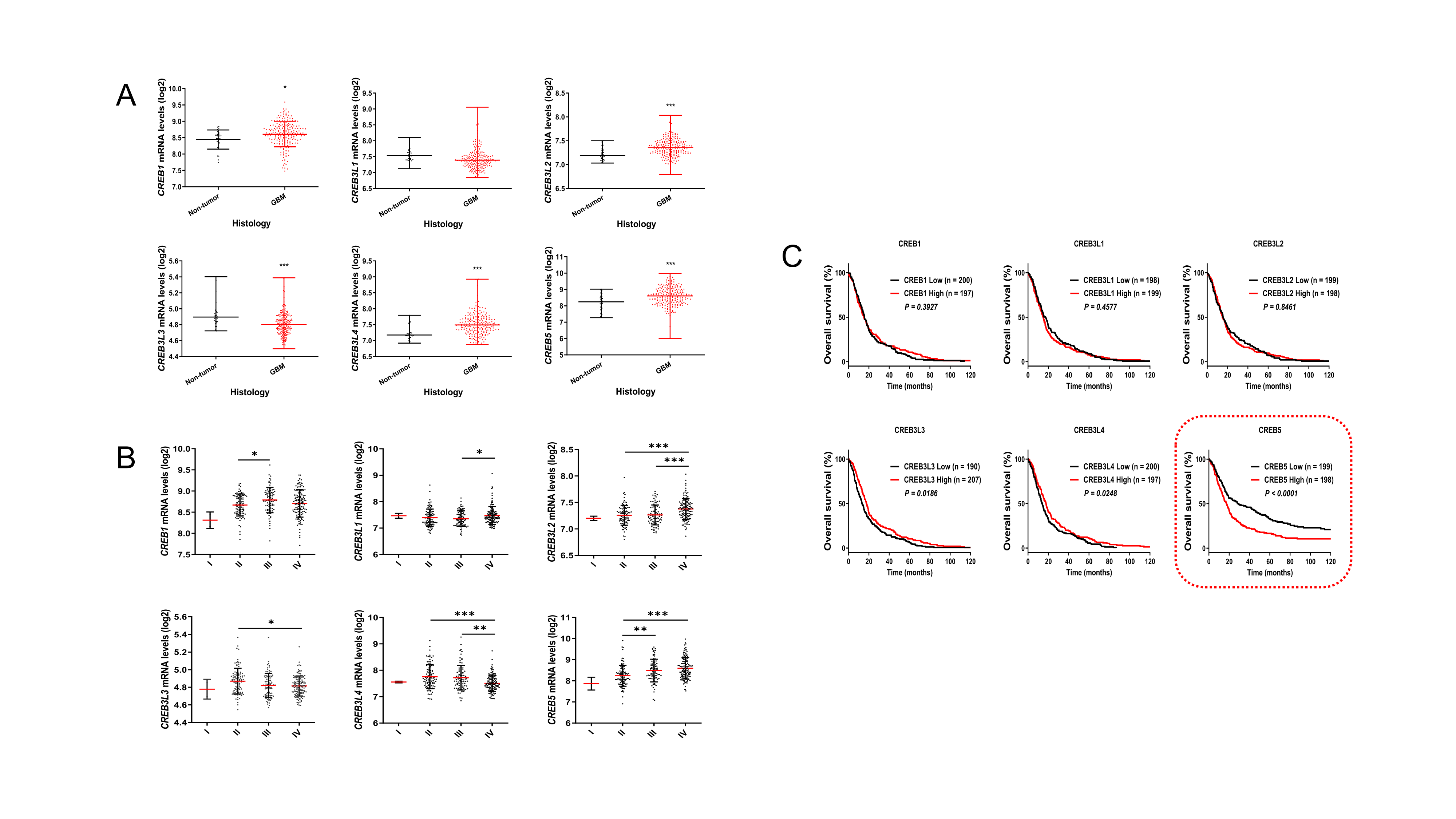

Supplement: Supplementary file 2 — Supplementary Figure 1 [file 41420_2024_1873_MOESM2_ESM.png]

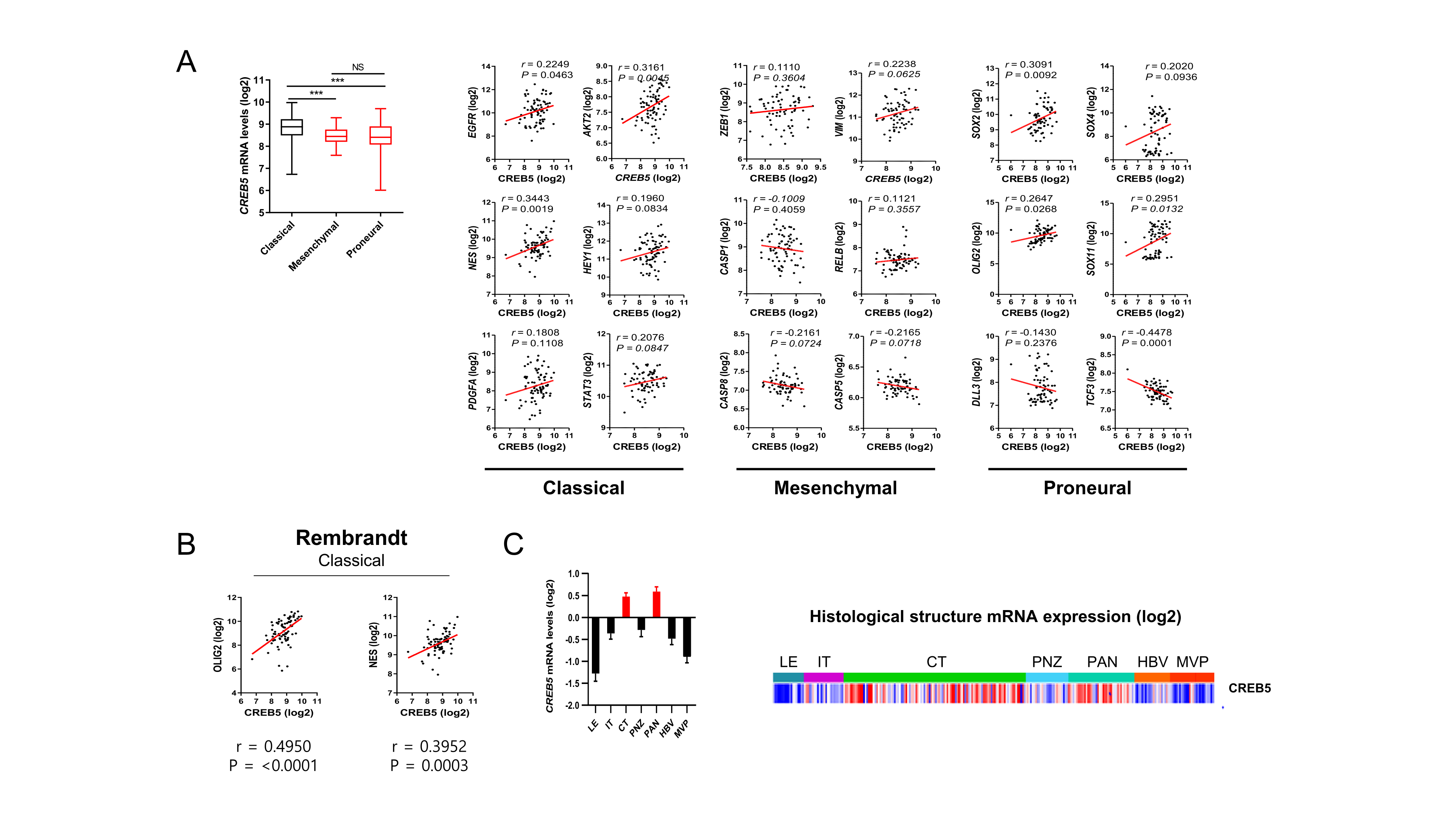

Supplement: Supplementary file 3 — Supplementary Figure 2 [file 41420_2024_1873_MOESM3_ESM.png]

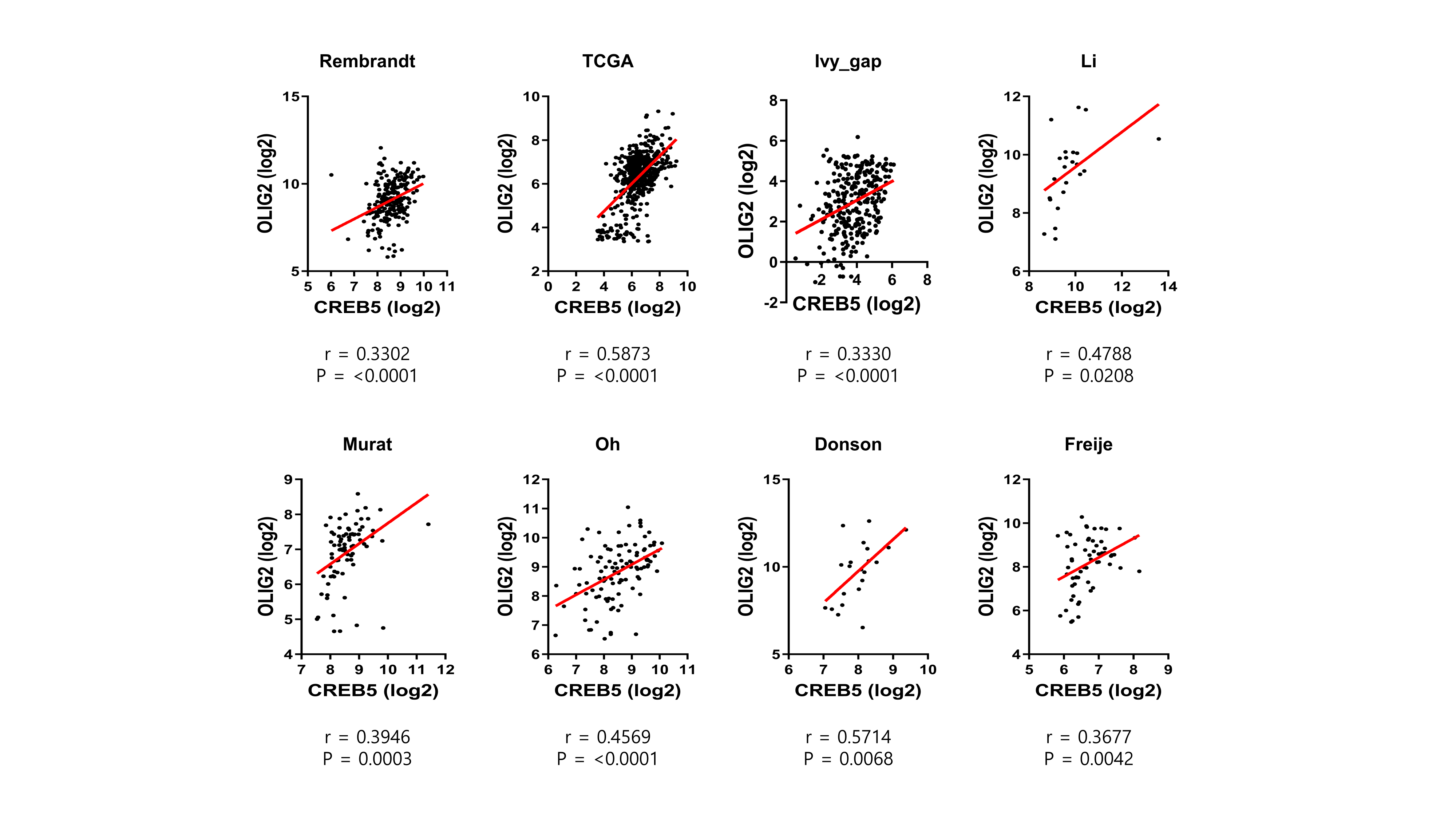

Supplement: Supplementary file 4 — Supplementary Figure 3 [file 41420_2024_1873_MOESM4_ESM.png]
