# Supplementary material for: CREB5 promotes the proliferation and self-renewal ability of glioma stem cells
Source: Cell Death Discov. 2024 Feb 28;10:103. doi: 10.1038/s41420-024-01873-z (PMC10901809; doi:10.1038/s41420-024-01873-z)

Original images

(Western blot)

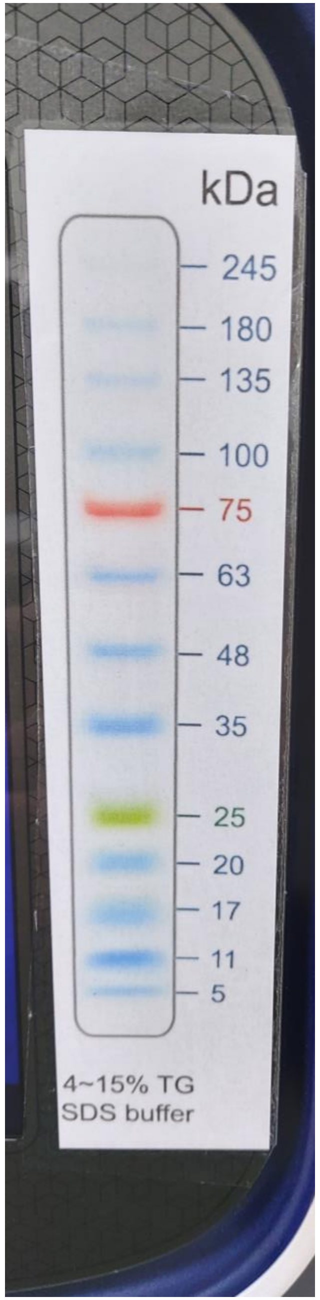

* All protein samples were prepared on the same day at the same time.

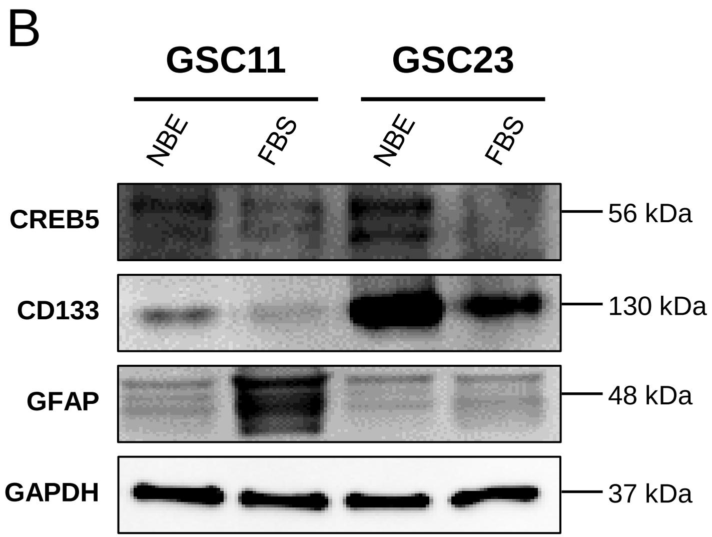

CREB5

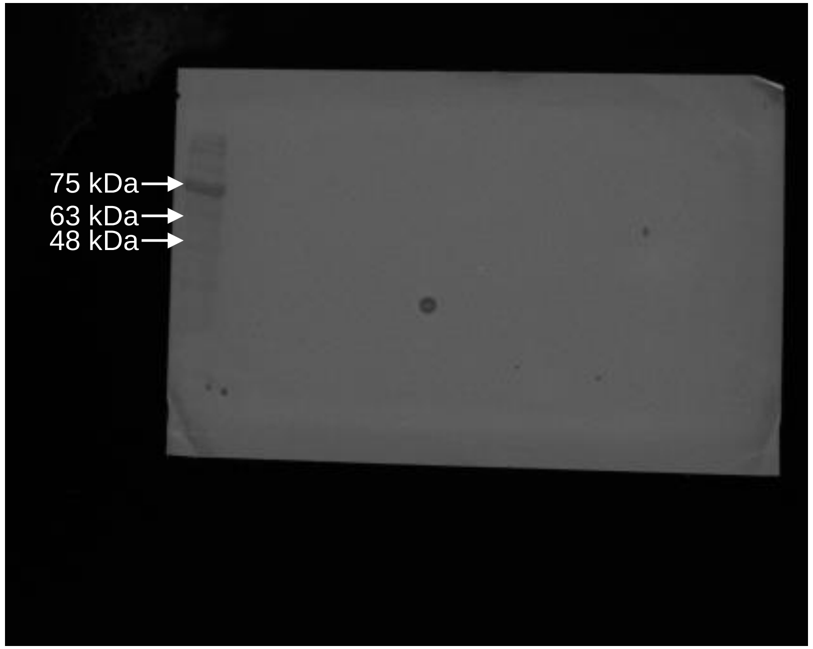

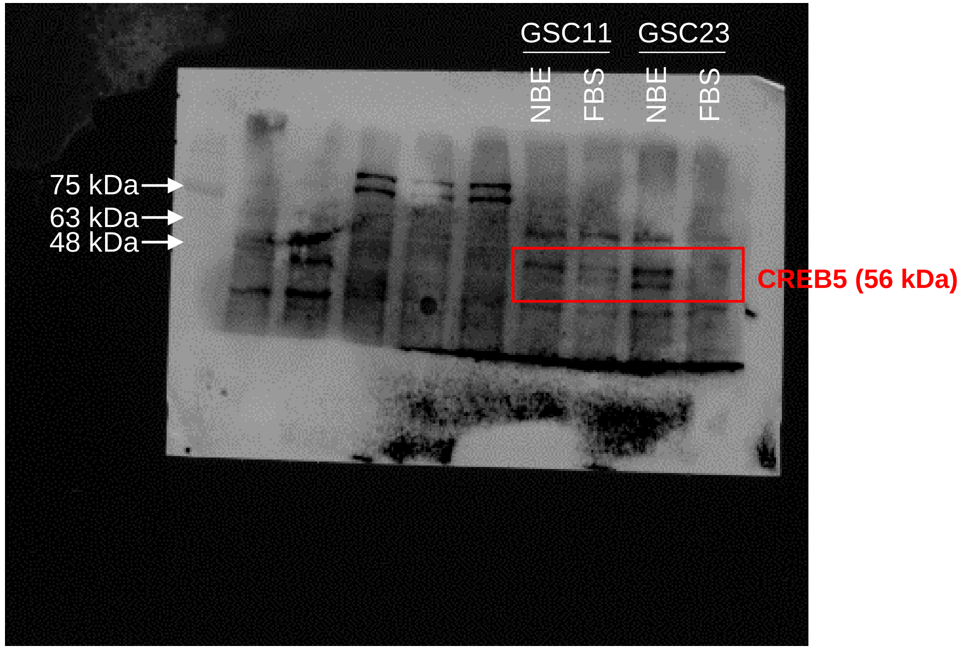

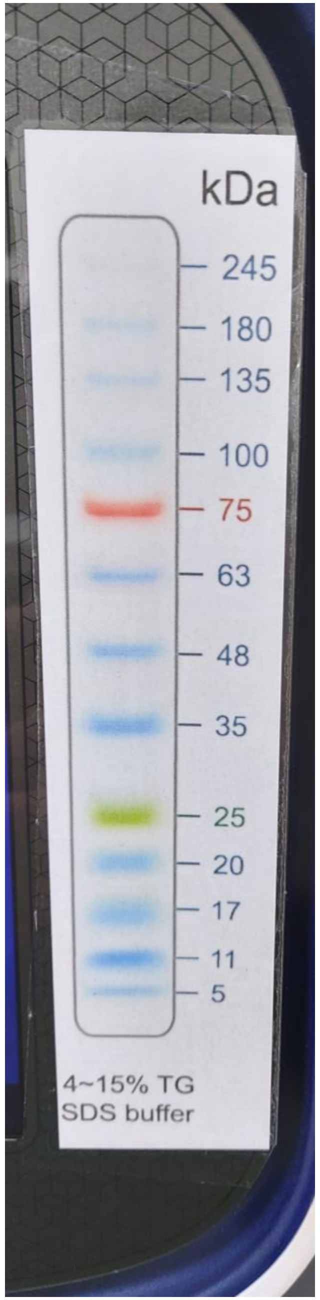

* All protein samples were prepared on the same day at the same time.

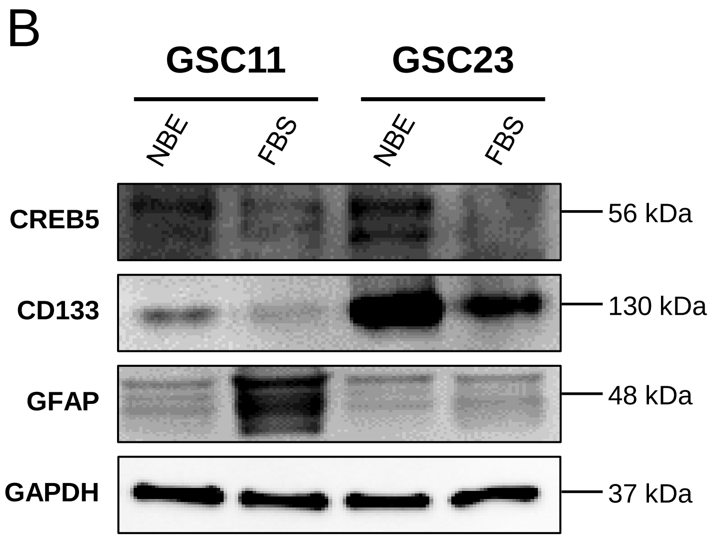

GAPDH

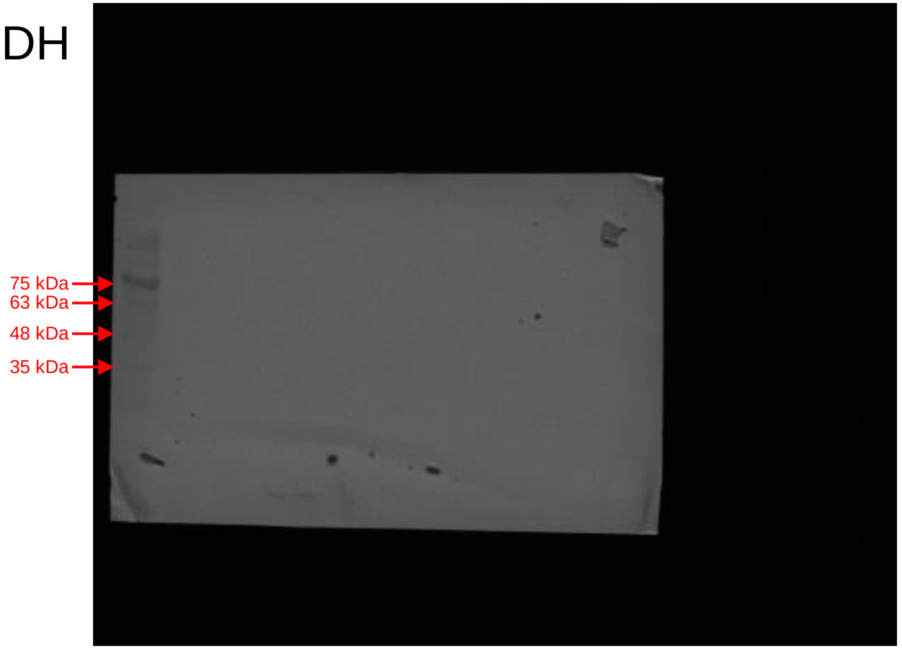

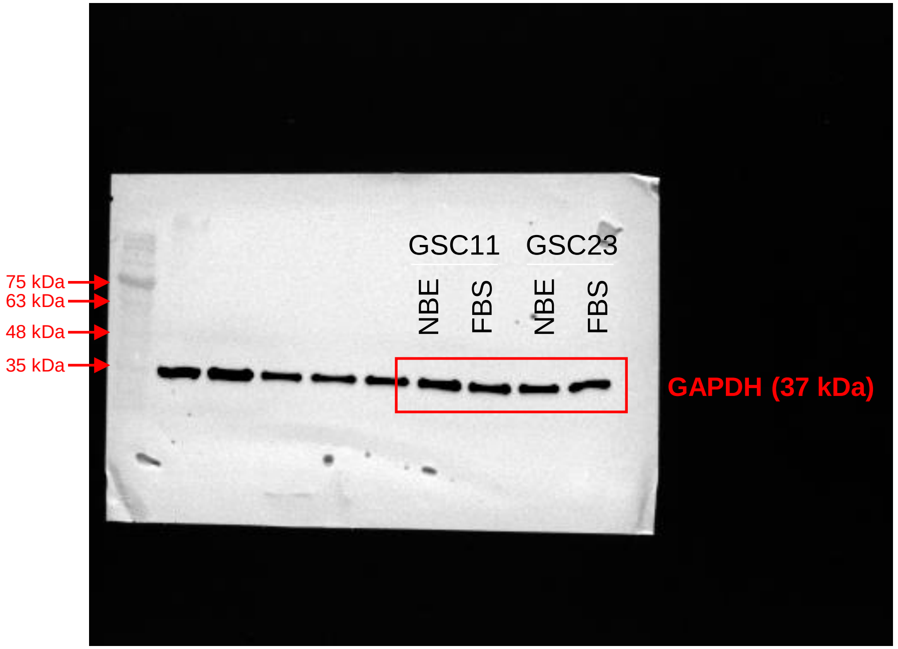

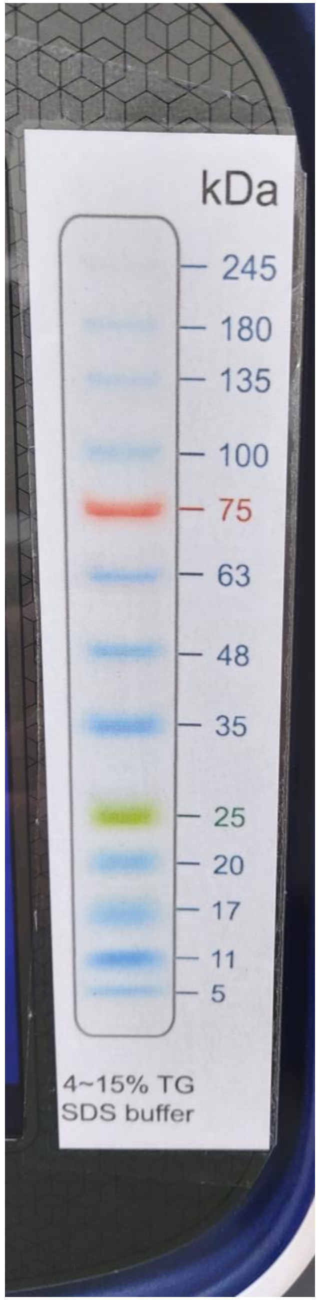

* All protein samples were prepared on the same day at the same time.

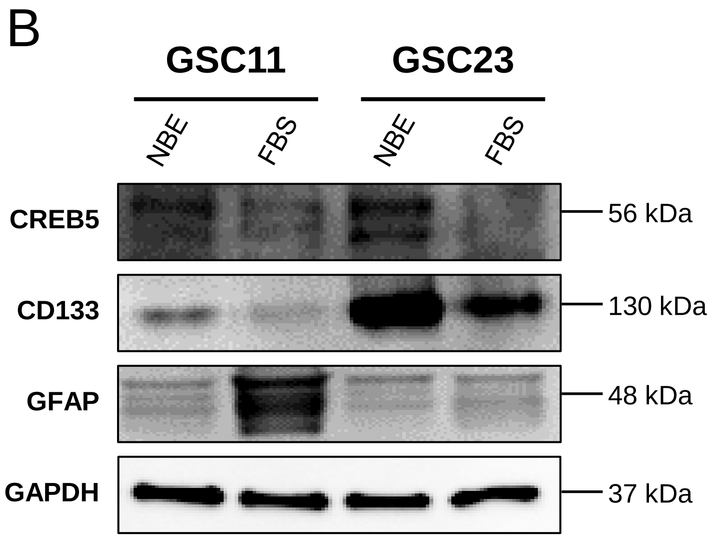

CD133

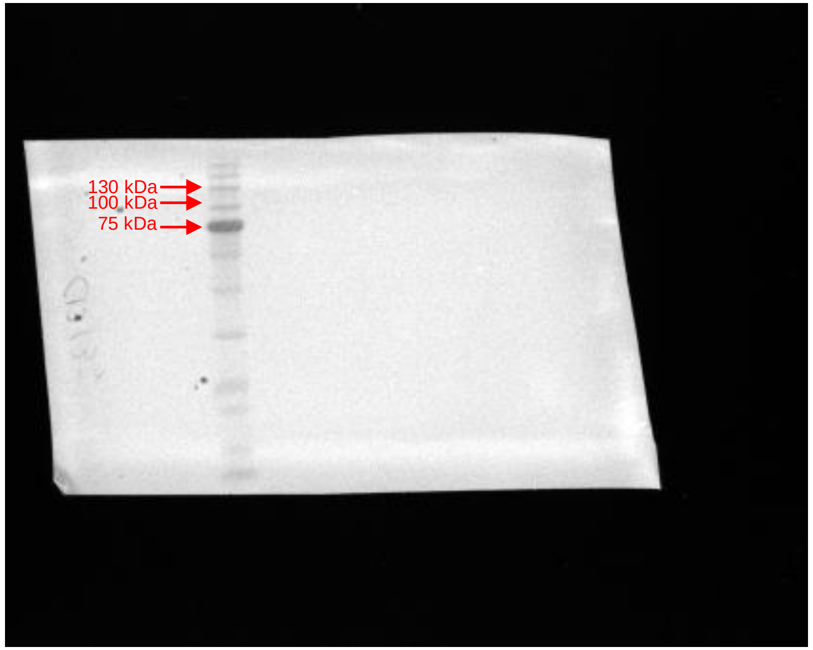

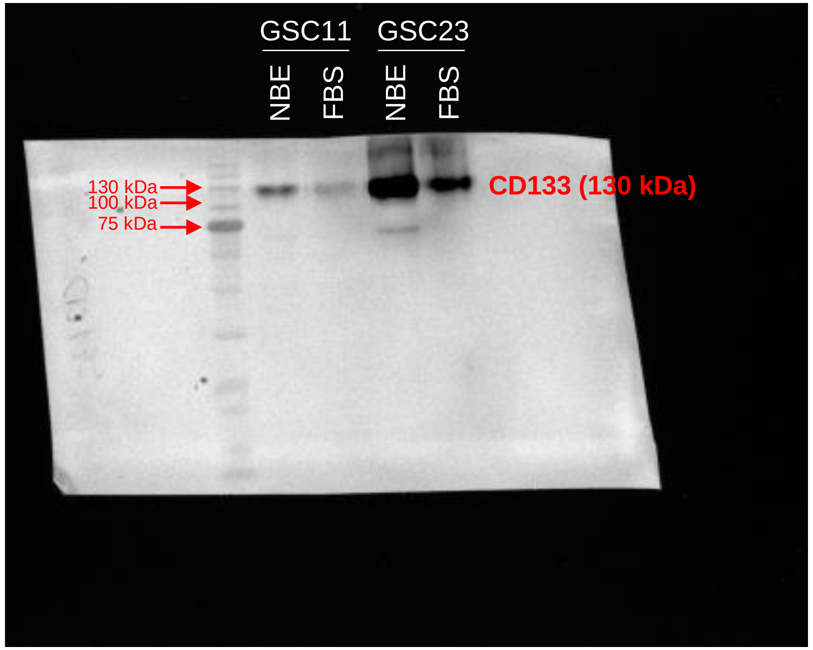

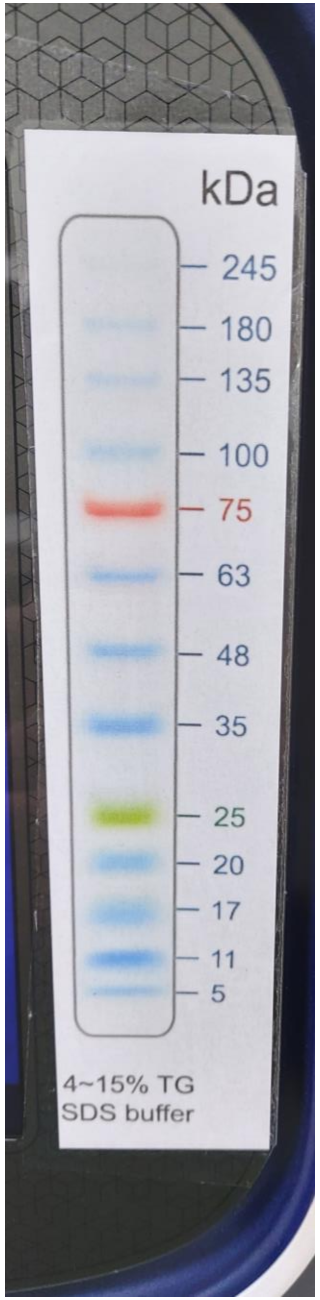

* All protein samples were prepared on the same day at the same time.

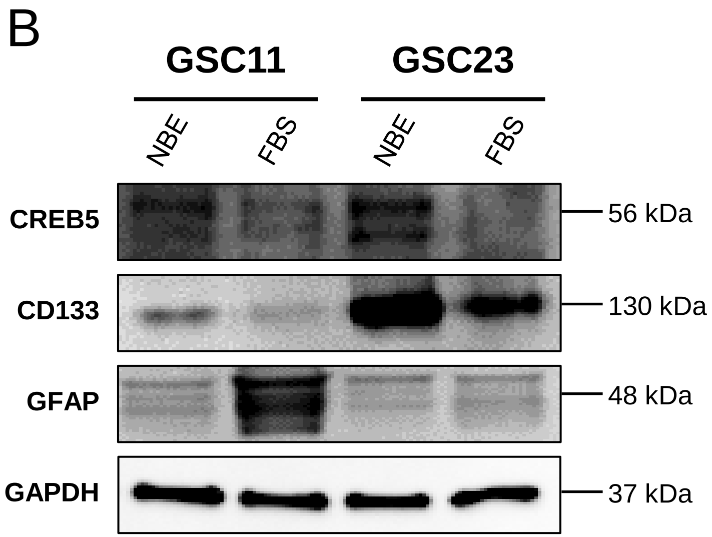

GFAP

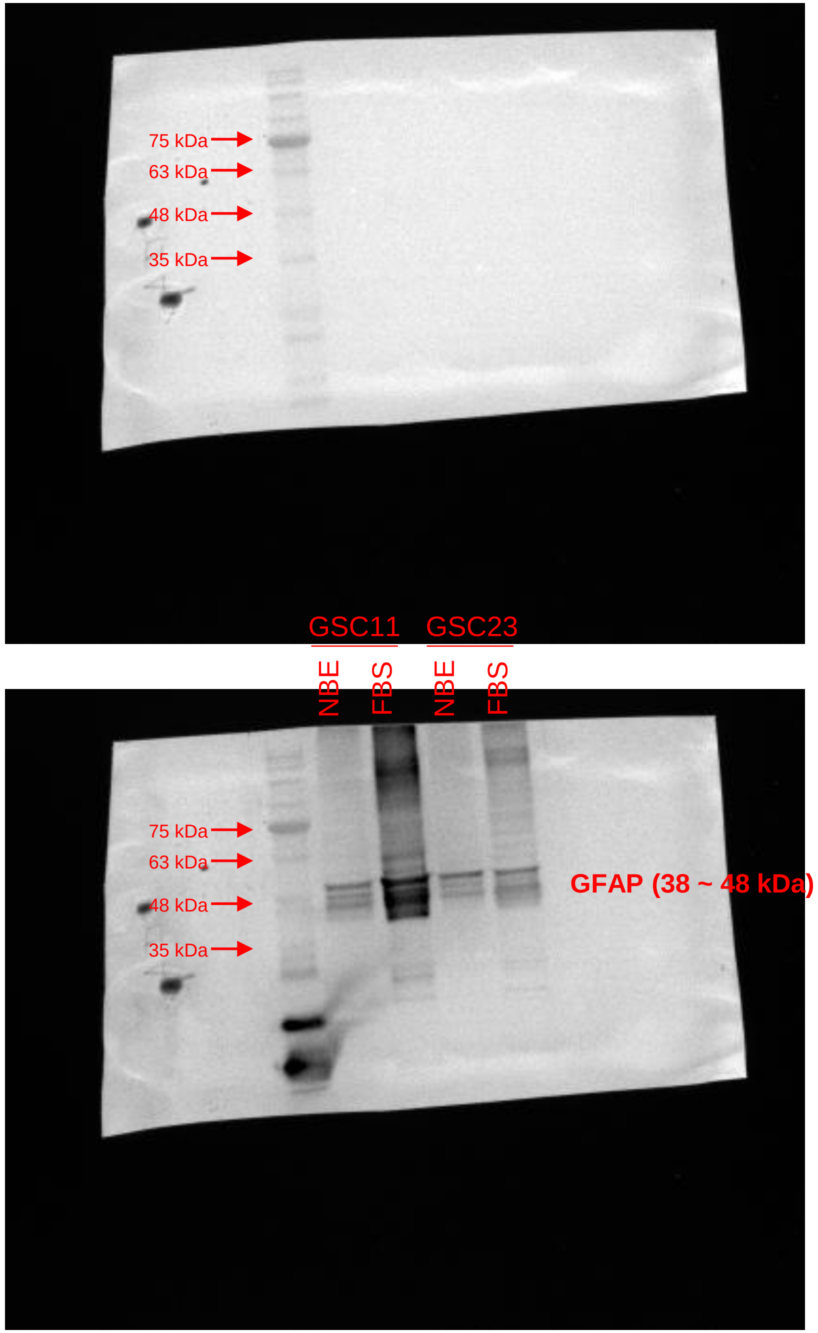

* All protein samples were prepared on the same day at the same time.

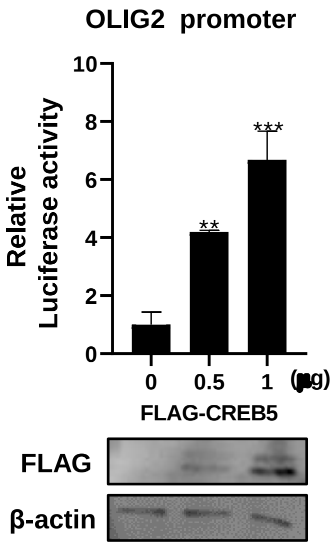

FLAG-CREB5

0 0.5 1.0 μg

FLAG

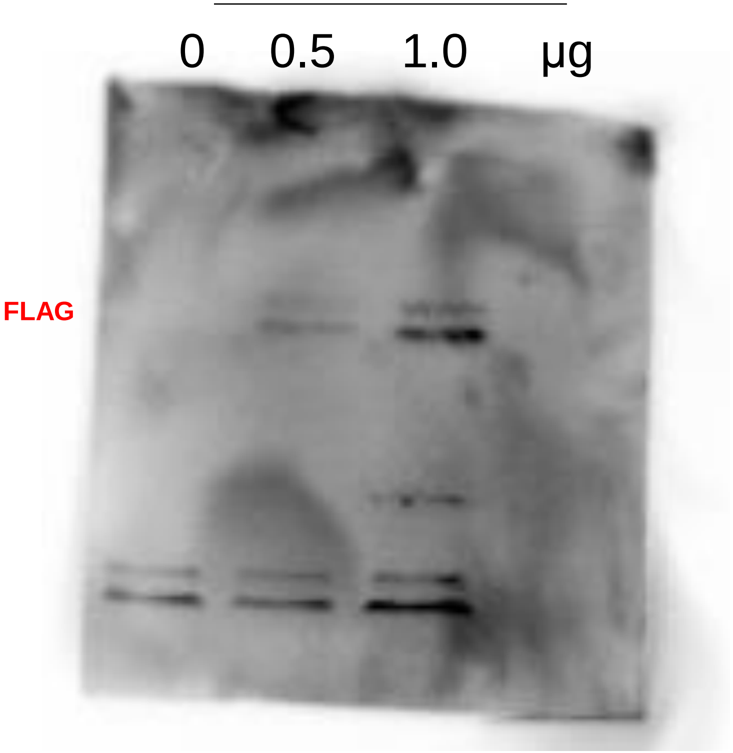

FLAG-CREB5

0 0.5 1.0 μg

β-actin (42 kDa)

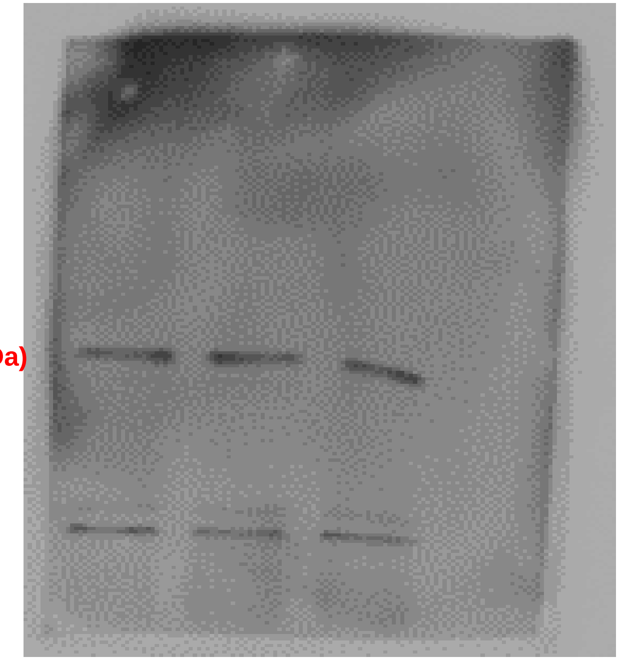

Supplement: Supplementary file 5 — Original Data File [file 41420_2024_1873_MOESM5_ESM.pdf]
